# Neuroimmune Tau Mechanisms: Their Role in the Progression of Neuronal Degeneration

**DOI:** 10.3390/ijms19040956

**Published:** 2018-03-23

**Authors:** Nicole Cortés, Víctor Andrade, Leonardo Guzmán-Martínez, Matías Estrella, Ricardo B. Maccioni

**Affiliations:** 1 Laboratory of Neurosciences, International Center for Biomedicine (ICC), Santiago 7630457, Chile; nicole.somnus@gmail.com (N.C.); victor.andrademf@gmail.com (V.A.); lguzmar@gmail.com (L.G.-M.); matiasestrella9@gmail.com (M.E.); 2 Faculty of Science, University of Chile, Santiago 7800020, Chile

**Keywords:** tauopathies, Alzheimer’s disease, tau protein, molecular networks, molecular functions, neuroimmunomodulation, inflammation

## Abstract

Progressive neurodegenerative pathologies in aged populations are an issue of major concern worldwide. The microtubule-associated protein tau is able to self-aggregate to form abnormal supramolecular structures that include small oligomers up to complex polymers. Tauopathies correspond to a group of diseases that share tau pathology as a common etiological agent. Since microglial cells play a preponderant role in innate immunity and are the main source of proinflammatory factors in the central nervous system (CNS), the alterations in the cross-talks between microglia and neuronal cells are the main focus of studies concerning the origins of tauopathies. According to evidence from a series of studies, these changes generate a feedback mechanism reactivating microglia and provoking constant cellular damage. Thus, the previously summarized mechanisms could explain the onset and progression of different tauopathies and their functional/behavioral effects, opening the window towards an understanding of the molecular basis of anomalous tau interactions. Despite clinical and pathological differences, increasing experimental evidence indicates an overlap between tauopathies and synucleinopathies, considering that neuroinflammatory events are involved and the existence of protein misfolding. Neurofibrillary tangles of pathological tau (NFT) and Lewy bodies appear to coexist in certain brain areas. Thus, the co-occurrence of synucleinopathies with tauopathies is evidenced by several investigations, in which NFT were found in the substantia nigra of patients with Parkinson’s disease, suggesting that the pathologies share some common features at the level of neuroinflammatory events.

## 1. Tau Protein the Context of Alzheimer’s Disease

Alzheimer’s disease (AD) is the most common type of brain dementia in aged populations (over 60 years old) [1], which gradually affects learning and memory as well as mood and behavior, displaying a constantly expanding prevalence and impact according to the World Health Organization (WHO). This expansive and epidemic behavior is concerning to medical and public health officials who are focusing their efforts on its prevention and treatment. In its biological context, two main etiological effectors have been reported: (i) Neurofibrillary tangles (NFT), composed by accumulation of the hyperphosphorylated protein tau, inside the neuron and assemblies of oligomeric structures denominated paired helical filaments (PHF) [2,3,4,5]; (ii) Senile plaques (SP), composed of deposits of the amyloid-β (Aβ) peptide of 39–42 aminoacidic residues, generated by the proteolytic excision of the amyloid precursor protein (APP) by the enzymes β and γ secretases, in the extracellular space, both promoting loss of synaptic processes and neuronal death [1,6]. Considering that tau protein is the major actor in the present discussion on the neuroimmune mechanisms leading to neuronal degeneration in tauopathies and AD, it is helpful to look at the origins of the discovery of this protein. Tau protein was discovered in two laboratories. Mark Kirschner’s group in October 1977 was the first to describe a protein, named as tau, the Greek letter to differentiate from tubulin [7]. Both proteins, tau and tubulin, showed similar electrophoretic migration in SDS gels due to similar molecular weights. Almost three months later, in January 1978, Maccioni and Seeds (1978) reported the same microtubule-associated protein after separating it from brain tubulin on the basis of cationic properties of tau. Thus, the novel microtubule-associated proteins (MAPs) showed to enhance both the rate of polymerization and the total amount of tubulin polymerized, which suggested its involvement in both the initiation and elongation of microtubules. This finding was simultaneously corroborated by studies on neuroblastoma cells [8]. 

**Figure 1:**
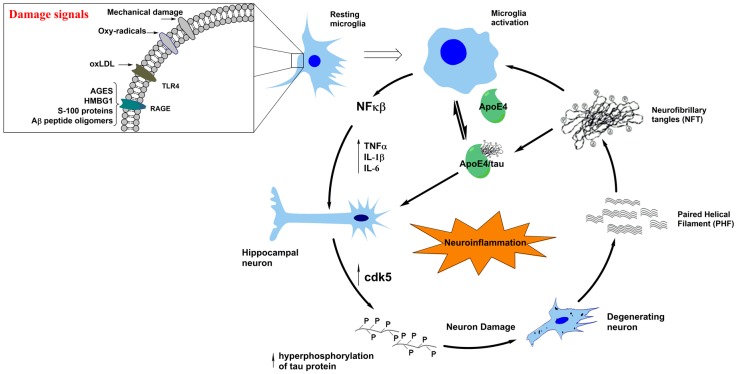
The potential mechanism involved in triggering tauopathies. “Damage signals” (inset at the upper left corner) sensitize resting microglia leading to an activated phenotype. This generates the over-release of increasing amounts of cytokines with the consequent effects on neuronal cells. There is activation of the protein kinase Cdk5, thus stimulating the Cdk5/p35 complex and tau hyperphosphorylation and neuronal degeneration. As a consequence of this processes, tau oligomers and PHFs are released to the extraneuronal environment, reactivating microglia via a positive feedback mechanism. This mechanism is stimulated by the ApoE4 protein. The reactivated microglia continues the cycle by increasing the levels of cytokines with the consequent effects on neuronal degeneration.

In the context of the neuroimmunomodulation hypothesis [9,10,11,12,13], we proposed that the onset of AD is mainly a consequence of the response of microglial cells to “damage signals” or tau oligomers (Figure 1), which trigger a neuro-inflammatory response, promoting an anomalous cascade of signaling that involves the release of the nuclear factor κB (NFκB), overproduction of pathological levels of cytokines and chemokines, and the consequent activation of neuronal receptors. This leads to an increase in the expression of the CDK5/p35 (cyclin-dependent kinase 5) complex, GSK3-β (glycogen synthase kinase 3-β), tau hyperphosphorylation, and the subsequent self-aggregation linked with neuronal degeneration [12]. Increasing evidence suggests that tau oligomers and polymers released upon neuronal apoptosis are capable of reactivating microglial cells, thus, favoring the continuous cascade of altered molecular signaling responsible for neuronal degeneration in tauopathies and AD [14]. 

## 2. Molecular and Structural Aspects of Tauopathies and AD

In neurons, there are several kinases that phosphorylate tau protein under physiological conditions and during AD, such as CDK5, GSK3-β, C-Jun-N-terminal kinase (JNK) that are regulated by cytokines released by astrocytes and microglia. CDK5 is a proline-directed serine-threonine kinase that phosphorylates serine and threonine residues, particularly serine 202 (Ser202) and threonine 205 (Thr205) residues of tau protein, also found in PHFs. CDK5 activity is regulated by p35 (and its split product p25) and p39, which have a short mid-life and phosphorylate CDK5 at its T-loop and translocate to the cellular membrane. This activation and translocation of CDK5 have important biological roles in cortex layer formation, neurite outgrowth, migration, the differentiation of neurons, synapse formation, and cognitive processes. CDK5 also regulates mitochondrial morphology and cell survival in response to stress [15,16,17]. GSK3-β is also a serine-threonine kinase, which phosphorylates tau at threonine 221 (thr221), and its kinase activity is upregulated by phosphorylation of tyrosine 216 (Tyr216) and tyrosine 279 (Tyr279) residues; meanwhile, serine-threonine kinase (Akt)-mediated phosphorylation of Ser9 and Ser21 residues reduce its activity. GSK3-β regulates memory processes by induction of LTD (long-term depression) and inhibition of LTP (long-term potentiation); these effects are reversed by insulin and Wnt, which inactivate GSK3-β. Also, GSK3-β promotes the assembly of actin to form filaments and the assembly of tubulin, leading to microtubule formation, thus regulating the reorganization of synaptic architecture [18,19,20]. Finally, JNK phosphorylates tau at serine 396 (Ser396) and threonine 221 (Thr221). This kinase has three isoforms that participate in brain development, immune modulation, induction of LTP, neurite formation, and JNK3, in particular, induces cell death by apoptosis [15].

At a molecular level, previous reports support the effect of AD on components that play roles in the glutamatergic synapse. Lee et al.’s experiments (2004) detected an unregulated overexpression of the metabotropic receptor 2 (Group 2) in patients with AD, which through extracellular signal-regulated kinases (ERK) receptors affect the abnormal hyperphosphorylation of tau protein observed in the disease [21]. Moreover, a recent study identified a functional role of the truncated extracellular C-terminal tau fragment in the hippocampus, promoting neuritic dystrophy, microtubules disorganization, a loss of mitochondria at nerve endings, and a decrease of pre-synaptic vesicular glutamate release by reduction of associated proteins [22]. Furthermore, recent evidence suggests that pathological tau also impairs synaptic transmission by the interaction of its N-terminal domain with synaptic vesicles, which restricts their normal mobilization and release of neurotransmitters, similar to the truncated extracellular C-terminal tau fragment. Disruption of the interaction of tau with vesicles is enough to rescue the affected synapses [23]. Among the different tau splice variants (oligomeric, fibrillary, or filamentous structures), extracellular forms have also been associated with other negative effects [24]. Briefly, Swanson and associates [24] found that 2N4R and 2N3R tau oligomers promote aggregation at the intracellular level, even more than monomers and fibrils or different oligomers from other tau isoforms. The effects were associated with invasion of tau into the somatodendritic compartment, affecting axonal fast transport through microtubule disassembly and changes in membrane organelles. 

The functional effects of tau in neuronal dysfunction have been corroborated recently in mice by using manganese-enhanced magnetic resonance imaging (MEMRI) [25]. Using similar approaches, more evidence appears from the effects of tau in vivo. PET (positron-emission tomography) imaging was used in patients with progressive supranuclear palsy (PSP), a human tauopathy usually lacking amyloid-β deposits. They also showed the presence of hyperphosphorylated tau in several regions, some previously related to consciousness, such as the striatum, thalamus, subthalamic region, midbrain, and cerebellar white matter [26]. This allows us to suggest the possible role of tau on interconnected conditions with AD since PSP presents behavioral and mood disorders. Furthermore, these kinds of pathologies have been associated with AD as part of its progression [27].

## 3. Neuroinflammation in Tauopathies

In the context of an integrative analysis of neuroimmune responses that affect tau in tauopathies, it is relevant to point out that neuroinflammation appears to be a common feature of several other degenerative disorders of the central nervous system. 

As seen in Figure 1, tau filaments and PHF released from degenerating neurons can trigger reactivation of microglial cells through a positive feedback mechanism. This allows the continuation of a vicious cycle of release of proinflammatory cytokines, activation of protein kinases at the neuronal level, and generation of anomalous tau polymers. These changes are also associated with the misfolding of tau and cytoskeleton disorganization [4,9,10]. Moreover, tau is implicated in more than 20 neurodegenerative diseases [23], and therefore, it is of interest to review at least some of them. On the previous context, new theories attempt to explain this pathology in complementary ways; there is enough evidence to relate behavioral disorders with the activation of neuroinflammatory processes as a pathway in AD progression [27]. In addition to AD, we will discuss how neuroinflammation modulates two other main tauopathies: frontotemporal dementia (FTD) and Parkinson’s disease (PD). As seen in Figure 1, the neuroinflammation pathway leads to neuronal damage, which has been widely observed in AD, but in addition, cumulated evidence supports its contribution to neurodegeneration in FTD and PD. Thus, neuroinflammation is proposed as a research focus for the treatment of tauopathies. 

Inflammation has been well documented in FTD. Previously, a study showed an increment in the levels of the pro-inflammatory cytokine tumor necrosis factor α (TNF-α) and the anti-inflammatory cytokine transforming growth factor β (TGF-β) in patients with a non-specified type of FTD, in comparison to normal controls. This suggests a possible role of inflammation in the pathogenesis of disease that was promptly confirmed by Bellucci et al., 2004 who demonstrated a robust evidence of high levels of pro-inflammatory cytokines interleukin-1 (IL-1) and cyclooxygenase-2 (COX2), along with activated microglia rounding cells, with tau inclusions in the brainstem and spinal cord of transgenic mice with tau mutation [28]. Moreover, this is correlated with cortex and hippocampus samples from a human postmortem brain carrying the same P301S mutation in tau gene [28,29,30]. Additionally, synaptic loss and microgliosis was observed before the NFT formation in the hippocampus of a transgenic mouse with the human tau mutation associated with the FTD model, thus, determining that inflammation can lead to NFT formation in FTD tauopathies [31], in agreement with our neuroimmunomodulation theory [9,10,13] (Figure 1). The microglial activation and inflammation process has been documented, also, in other transgenic mice with tau mutation model of FTD, demonstrating further that these alterations were dependent on tau expression [32]. Furthermore, the neuroinflammatory process has been proposed as a potential diagnostic tool through the in vivo evaluation of microglial activation, using DLB imaging with the translocator protein (TSPO) ligand [^11^C]-PK11195 in FTD and other tauopathies [33,34,35].

## 4. A Typical Tauopathy: Frontotemporal Dementia (FTD)

Frontotemporal dementia (FTD) is a heterogeneous syndrome that includes a wide spectrum of disorders, overall affecting the zones of the frontal and temporal lobe in the human brain, which cause language, motor, and behavioral alterations [36,37]. FTD is the second most important “dementia” after AD, in terms of the number of people that suffer from the disease [38,39]. In general, FTD affects men and women in similar proportions, and it starts in individuals at 45–65 year-of-age. They have an expected range of survival of two to 20-years from the onset, with an average of eight years [40,41,42]. FTD has a prevalence of 3.6–9.4 people affected per 100,000, varying according to the age of onset [40,42,43]. Although literature shows diverse nomenclature in classifying FTD, we consider the terminology used in the most recent updates that go deep into different FTD subtypes, which is concordant with a previous clinical classification, establishing three main forms of FTD, namely: (a) the behavioral variant of FTD (bvFTD), (b) the non-fluent variant (nfFTD), and (c) the semantic variant (svFTD). The latter two are categorized as primary progressive aphasias (PPA) given that they primarily affect language functions according to clinical diagnosis criteria. Of the three forms, the most common type is bvFTD, which encompasses around 60% of cases [37,42,44]. Furthermore, other alterations in FTD categorized as “related FTD” include: motoneuron disorders FTD (MNFTD), progressive supranuclear palsy (PSP-FTD), and corticobasal syndromes (CBS) [37,45]. Finally, there is another distinction based on the neuropathological alterations that affect mainly the frontal and temporal lobes, named “frontotemporal lobar degeneration” (FTLD), where specific protein aggregates determine different types of FTD termed: FTLD-tau (tau protein), FTLD-TDP (transactive response of DNA-binding protein), FTLD-FET (FUS, EWS, and TAF15 protein family), and FTLD-UPS (Ubiquitin Proteasome System) [37,46,47]. These groupings are not mutually exclusive, and we will focus here on the FTD caused by tau inclusions (FTD-tauopathies), which encompass around half of the total FTDs and are characterised by the presence of tau aggregates in neuronal and/or glial cells [48]. These include Picks disease as a classic definition (PiD-FTD), CBS, and PSP-FTD. In addition, there are other less common FTD-tau, known as globular glial tauopathies (GGT) and argyrophilic grain disease (AGD), that are not framed in the previous divisions [37,47,49]. These last two, together with CBP and PSP FTD tauopathies, are predominantly formed by 4R tau repetitions, while PiD-FTD tau is mainly associated with 3R tau aggregate [49].

Besides the inflammatory detection in FTD, PET techniques using tau radioligands have recently shown potential as a specific biomarker of FTD, given that [^18^F] AV-1451 was abnormally distributed in patients with bvFTD caused by a specific mutation in the tau gene. This, in comparison to healthy subjects, besides the [^18^F] THK-5351 and the [^11^C] PBB3 radioligands that showed high selectivity in PSD-FTD, and an affinity for a wide range of tauopathies respectively, has become a promising tool of specific diagnosis in FTD [50,51,52]. Additionally, cerebrospinal fluid (CSF) biomarkers established by the specific ratio between total levels of tau and Aβ_1–42_, and phosphorylated tau and Aβ_1–42_ has been described as a robust discriminator between FTD and AD [53,54].

Until the present time, there has been no approved treatment to cure or prevent FTD by the FDA, but the behavioral alterations have been positively attenuated using different selective serotonin reuptake inhibitors (SSRIs), as described below [55]. Citalopram, an antidepressant with high selectivity against the serot1rgic system, was effective in improving disinhibition, irritability, and depression in patients with FTD and was capable of reversing the effect over affected areas associated with disinhibition in FTD subjects [38,56]. Moreover, Trazodone, a drug that increases the extracellular serotonin (5-HT) levels in the frontal cortex, has shown a decrease in the irritability, agitation, depressive symptoms, and eating disorders previously enhanced in a group of patients with FTD [57]. The serotoninergic modulation to improve the behavioral alterations in FTD-tauopathies is in agreement with our recently postulated hypothesis, which suggest that alterations in the dopaminergic pathway together with serotonin depletion are implicated in the initial events of the pathogenesis of AD, leading to late-onset depression and posterior triggering of disease [27].

On the other hand, promising advances have been obtained, focused in the tau-related process to treat diverse tauopathies, highlighting inhibitors of tau and phosphorylated tau, stabilizers of microtubules, and tau anti-aggregating molecules [45]. TRx037, a bioavailable inhibitor of tau aggregation has demonstrated beneficial properties in bvFTD and recently gave auspicious results in AD patients through diverse clinical trials [58].

## 5. Molecular Interactions and the Links between Tauopathies and Parkinson’s Disease

Parkinson’s disease (PD) is the second most common neurodegenerative disease after Alzheimer’s disease (AD). It is a movement disorder whose etiopathogenesis involves a combination of genetic and environmental factors. The precise molecular basis remains unclear. Although the initial causes of PD are not clearly determined, factors such as aging, oligomerization of α-synuclein (α-syn), mitochondrial dysfunction, oxidative stress, and neuroinflammation appear to play a pathogenic role in this disease [59]. The prominent neuropathological manifestation of PD is the degeneration of neurons containing neuromelanin in substantia nigra pars compacta, resulting in a loss of dopamine and the presence of cytoplasmic inclusions of proteins, called Lewy Bodies (LB), composed mainly of filaments of α-syn [60]. α-Syn is a protein of 140 amino acids with three distinct regions. The amino-terminal end is positively charged, the central hydrophobic segment, between residues 61–90 (also called the non-amyloid component or NAC), and the carboxyl end, which is negatively charged. It is a lipid binding protein that possesses four tyrosine residues, one (Tyr39) near the amino terminus and three tyrosines (Tyr125, Tyr133, and Tyr136) near the carboxyl terminus [61].

At present, approximately 2% of the population over 50 years of age is affected by PD [62]. The most common clinical signs of PD can be divided into motor, cognitive (dementia), neuropsychiatric (depression and anxiety), and autonomic dysfunctions (hypotension and constipation). In the case of motor alterations, the following clinical signs stand out: (i) rest tremor, (ii) bradykinesia (slow movement, especially of complex voluntary movements), (iii) postural instability, and (iv) rigidity [63].

The loss of dopaminergic neurons in the substantia nigra produces a decrease in dopamine levels in the striatum, generating deregulation of the circuits of the basal ganglia, which leads to the appearance of motor symptoms. In summary, PD belongs to a group of neurodegenerative disorders called synucleinopathies, which includes Parkinson’s disease with dementia (PDD), Lewy body dementia (DLB), and multiple system atrophy (MSA). DLB symptomatology is characterized by generating parkinsonism, hallucinations (mostly of visual character), and dementia [64]. These symptoms make it difficult to make a diagnosis of this disease, so there is a criterion that allows approaching the diagnosis of DLB. This is to verify that the patient has at least two or more of the symptoms mentioned for this disease [64].

The α-syn hyperphosphorylations lead to protein misfolding and its subsequent oligomerization. These α-syn deposits are ubiquitous in the central nervous system, especially in the terminals of the presynaptic neurons. This misfolding and oligomerization of α-syn is called synucleinopathy [65,66]. PD and tauopathies are certainly caused by two different types of aggregates, synuclein in the case of PD and tau assemblies in the case of tauopathies. However, there is co-occurrence of synucleinopathies with tauopathies and also with other diseases of protein misfolding, and they are frequent. Both neurodegenerative diseases suggest interactions of pathological proteins that enter common pathogenic pathways, although the etiology of most of these processes remains elusive [67,68,69].

Despite clinical, pathological, and genetic differences, increasing experimental evidence indicates an overlap between tauopathies and synucleinopathies. NFT and LB neurons often coexist in the brain or even within the same cell [70,71]. This co-occurrence of synucleinopathies with tauopathies is evidenced by findings of several investigations, including those of Schneider et al., 2006, in which NFT were found in the substantia nigra of patients with PD associated with displacement damage [72]. In turn, Joachim et al., 1987, through immunolabeling, found the presence of NFT in the substantia nigra of patients with AD, Down Syndrome, and PD associated with AD [73]. Phosphorylated tau has also been seen in dopaminergic neurons of individuals with PD and PDD [74]. In turn, in studies performed on a transgenic model that overexpressed human α-syn, phosphorylated tau was also found in striatal neurons [75]. Other authors observed that by suppressing α-syn expression, no phosphorylation of GSK-3β occurs [76]. Thus, the mechanism that relates tau to α-syn can be explained as follows: when an increase in α-syn expression, and consequently, an increase of this protein in the brain is generated, phosphorylation of GSK-3β occurs that allows this protein kinase to phosphorylate to tau [77]. Once tau phosphorylation is potentiated, NFT begins to form in the individual. LB has been observed in approximately 60% of AD patients, both in familial and sporadic forms [70,78]. This overlapping of tauopathies and synucleinopathies is associated with a more aggressive progression of the disease and an accelerated cognitive dysfunction [79,80,81]. This may suggest that Aβ, tau, α-syn, and activated GSK-3β would interact synergistically, promoting their oligomerization and accumulation among themselves [82,83].

In vitro studies have shown binding of α-syn to tau, inducing their phosphorylations [84]. The α-syn induces tau fibrillary formation, and the coincubation of both proteins synergistically promotes the mutual formation of pathological filaments [82]. In vivo evidence of an interaction between α-syn and tau has been observed in mice that overexpress Ala53Thr synuclein α (A53T SNCA), demonstrating positive inclusions for both markers [82]. In turn, Muntane and colleagues [85] found phosphorylated tau in the amino acid residue Ser396 in the fractions enriched in PD cortex synapses [85], whereas phosphorylated tau (at Ser202 and Ser396/404) was observed in the brainstem of mice overexpressing A-309P α-syn [86]. Further evidence of direct tau and α-syn involvement in these pathologies is supported by the induction of α-syn as related to tau hyperphosphorylation, in the 1-methyl-4-phenyl-1,2,3,6-tetrahydropyridine (MPTP) model of parkinsonism in mice [77] and co-localization of phosphorylated tau and α-syn in both NFT and LBs [87]. α-syn oxidatively modified by the proteasome promotes the recruitment of tau to protein inclusions in oligodendroglial cells in synucleinopathies [88]. Moreover, an in vitro study showed that α-syn promotes phosphorylation of tau in the amino acid residues Ser262 and Ser356 by protein kinase A (PKA) [84]. It is noteworthy that PKA does not phosphorylate tau residues Ser396 and Ser404, whereas GSK3-β does not phosphorylate tau residue Ser262, which could suggest that both kinases have a synergistic role in the induction of a α-syn-mediated tauopathy. Other studies found that GSK3-β was activated in an α-syn dependent manner, which hyperphosphorylates tau at residues Thr181, Ser396 and Ser404 [77,89,90,91]. This effect appears to be the result of both an increase in the activity of GSK3-β [89,92] and the formation of a tripartite complex between GSK3-β, α-syn, and tau. However, GSK3-β is not the only kinase that binds to α-syn and to hyperphosphorylated tau. In fact, activation of ERK and JNK, which also phosphorylate tau in Ser396 and Ser404, correlate with the presence of phosphorylated tau in mouse transgenic models, which overexpress α-syn [86,93,94]. Interestingly, by using fluorescence intensity distribution analysis (FIDA), Nübling and his collaborators [95] have shown that tau and α-syn can form co-oligomers, and that coaggregation occurs even at nanomolar concentrations but only in the presence of cationic aggregation inducers such as Al^3+^ and Fe^3+^ or DMSO [95]. On the other hand, tau phosphorylation by GSK3-β strongly increased the formation of mixed oligomers [95]. These observations demonstrate that tau accelerates α-syn polymerization, and that α-syn can act as an inducer of tau polymerization through its hydrophobic NAC region. In this perspective, a major difference between tau and α-syn is that α-syn is prone to self-aggregate, whereas tau cannot aggregate by itself and requires an inducing agent [96].

## 6. Conclusions

Neuroimmune mechanisms directly involved in AD are also part of several disorders of tau protein or tauopathies. According to our neuroimmunomodulation theory, alterations in the cross-talks between glial cells and neurons as a consequence of the activity of damage signals, e.g., iron overload, vitamin B deficiencies, Aβ peptide, and also tau oligomers released to extracellular media, [14] trigger the production of proinflammatory cytokines that finally affect neurons by activating the protein kinases CDK5 and GSK3-β, with the consequent tau hyperphosphorylations and aggregation into pathological PHFs and NFTs (Figure 1). A relationship between tau modifications and protein misfolding with synucleinopathies involved in PD and LBD has also been postulated. On the other hand, mood and behavioral disorders seem to be prodromal manifestations prior to neuroinflammatory signaling at the level of the hippocampus. The stages of consciousness in relation to tauopathies involving alterations in the frontal lobe and sub-cortical regions, including the thalamus, seem to be affected as the inflammatory damage spreads. These phenomena can give us insights into different disorders that could be related to the progression of these neurodegenerative disorders.

## Abbreviations

AD	Alzheimer’s disease
WHO	World Health Organization
NFT	Neurofibrillary tangles
PHF	Paired helical filaments
SP	Senile plaques
Aβ	Amyloid-β
APP	Amyloid precursor protein
MAP	Microtubule-associated proteins
NFκB	Nuclear factor κB
CNS	Central nervous system
EEG	Electroencephalogram
NMDA	*N*-methyl-d-aspartate
CDK5	Cyclin-dependent kinase 5
GSK3-β	Glycogen synthase kinase 3-β
JNK	c-Jun-*N*-terminal kinase
Akt	Serine/threonine kinase
LTD	Long-term depression
LTP	Long-term potentiation
ERK	Extracellular signal-regulated kinases
MEMRI	Manganese-enhanced magnetic resonance imaging
PET	Positron-emission tomography
PSP	Supranuclear palsy
FTD	Frontotemporal dementia
bvFTD	Behavioural variant of Frontotemporal dementia
nfFTD	Nonfluent variant of Frontotemporal dementia
MNFTD	Motoneuron disorder
PSP	Progressive supranuclear palsy
svFTD	Semantic variant of Frontotemporal dementia
CBS	Corticobasal syndromes
FTLD	Frontotemporal lobar degeneration
TDP	Transactive response of DNA-binding protein
FET	FUS, EWS, and TAF15 protein family
UPS	Ubiquitin Proteasome System
PiD	Picks disease
GGT	Globular glial tauopathies
AGD	Argyrophilic grain disease
C9ORF72	Chromosome 9 open reading frame 72
TNF-α	Tumor necrosis factor α
TGF-β	Transforming growth factor β
IL	Interleukin
COX2	Cyclooxygenase-2
TSPO	Translocator protein
CSF	Cerebrospinal fluid
5-HT	Serotonin
SSRIs	Selective serotonin reuptake inhibitors
PD	Parkinson’s disease
α-syn	α-Synuclein
LB	Lewy Bodies
NAC	Non-amyloid component
PDD	Parkinson’s disease with dementia
DLB	Lewy body dementia
MSA	Multiple system atrophy
MPTP	1-methyl-4-phenyl-1,2,3,6-tetrahydropyridine
A53T SNCA	Ala53Thr synuclein α
PKA	Protein Kinase A
FIDA	Fluorescence intensity distribution analysis
